# Exploring Sphingolipid Implications in Neurodegeneration

**DOI:** 10.3389/fneur.2020.00437

**Published:** 2020-05-21

**Authors:** Alice V. Alessenko, Elisabetta Albi

**Affiliations:** ^1^Emanuel Institute of Biochemical Physics, Russian Academy of Sciences, Moscow, Russia; ^2^Department of Pharmaceutical Science, University of Perugia, Perugia, Italy

**Keywords:** sphingolipids, neurodegeneration, diagnostic marker, therapeutic target, brain

## Abstract

Over the past decade, it was found that relatively simple sphingolipids, such as ceramide, sphingosine, sphingosine-1-phosphate, and glucosylceramide play important roles in neuronal functions by regulating rates of neuronal growth and differentiation. Homeostasis of membrane sphingolipids in neurons and myelin is essential to prevent the loss of synaptic plasticity, cell death and neurodegeneration. In our review we summarize data about significant brain cell alterations of sphingolipids in different neurodegenerative diseases such as Alzheimer's disease, Parkinson disease, Amyotrophic Lateral Sclerosis, Gaucher's, Farber's diseases, etc. We reported results obtained in brain tissue from both animals in which diseases were induced and humans in autopsy samples. Moreover, attention was paid on sphingolipids in biofluids, liquor and blood, from patients. In Alzheimer's disease sphingolipids are involved in the processing and aggregation of β-amyloid and in the transmission of the cytotoxic signal β-amyloid and TNFα-induced. Recently, the gangliosides metabolism in transgenic animals and the relationship between blood sphingolipids changes and cognitive impairment in Alzheimer's disease patients have been intensively studied. Numerous experiments have highlighted the involvement of ceramide and monohexosylceramide metabolism in the pathophysiology of the sporadic forms of Parkinson's disease. Moreover, gene mutations of the glucocerebrosidase enzyme were considered as responsible for Parkinson's disease via transition of the monomeric form of α-synuclein to an oligomeric, aggregated toxic form. Disturbances in the metabolism of ceramides were also associated with the appearance of Lewy's bodies. Changes in sphingolipid metabolism were found as a manifestation of Amyotrophic Lateral Sclerosis, both sporadic and family forms, and affected the rate of disease development. Currently, fingolimod (FTY720), a sphingosine-1-phosphate receptor modulator, is the only drug undergoing clinical trials of phase II safety for the treatment of Amyotrophic Lateral Sclerosis. The use of sphingolipids as new diagnostic markers and as targets for innovative therapeutic strategies in different neurodegenerative disorders has been included.

## The Dark World of the Brain and Sphingolipids

**Figure 1:**
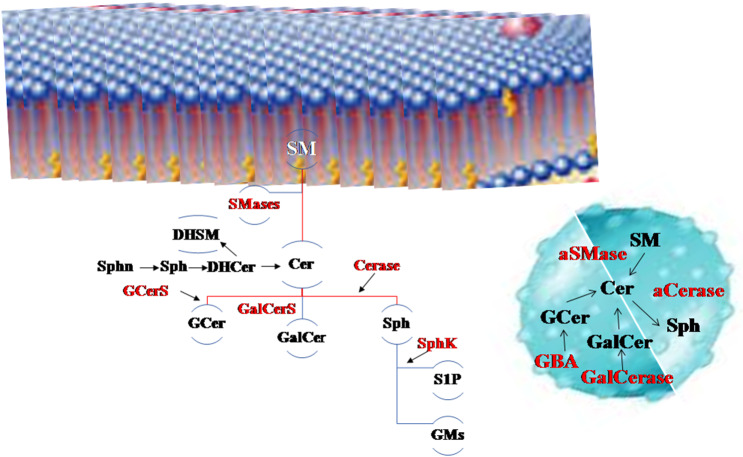
The main sphingolipids (SphLs) and metabolism SphL enzymes in neurodegeneration. Above, the cell membrane, on the right the lysosome. Sphingomyelinase (SMase) degrades sphingomyelin (SM) to ceramide (Cer) and it is catabolized by ceramidase (Cerase) to sphingosine (Sph) that is utilized to form sphingosine-1-phosphate (S1P) by sphingosine kinase (SphK) and gangliosides (GMs). Cer is also used to form glucosylceramide (GCer) by glucosylceramide synthase (GCerS) and galactosylceramide (GalCer). GCer is degraded by glucocerebrosidases (GBA) and GalCer by galactosylceramidase (GalCerase). Also precursors of Cer as sphinganin (Sphn), sphingosine (Sph) and dihydroceramide (DHCer) are present in the brain as well dihydrosphingomyelin (DHSM).

In the last 20 years the face of sphingolipids (SphLs) has changed. It took a long time to understand that these lipids are not only structural membrane molecules with a stiffening role but they are functional molecules fundamental for cell fate. Today, SphLs have great interest in brain physiopathology and therefore in the entire body considering that in humans the brain controls their own physical and inner life and allows relations with the outside world. In fact, specific areas of the brain control movement, sensitivity, vision, listening, thought, word, emotions, music and others, but all areas are rich in interconnections and molecular interactions. Today, mechanisms that underlie the functions of neuronal and glial cells are not completely clarified and many studies highlight that SphLs are some of the main actors (1). In the brain, SphLs play crucial roles by regulating the rate of growth, differentiation, and death of central nervous system (CNS) cells. Violation of the balance of the different classes of SphL leads to changes in the fate and functions of neuronal cells. Now there is a plethora of information on SphL metabolism to which contrasting roles are attributed. The most involved SphLs in neurodegeneration are simple molecules such as sfingosines (sphinganin, Sphn; sphingosine, Sph; sphingosine-1-phosphate, S1P) and ceramide (Cer), derived molecules as glucosylceramide or cerebroside (GCer) and galactosylceramide (GalCer), and sphingomyelin (SM), and finally more complex molecules as gangliosides (GM, glicolipids with sialic acid residues). Moreover, relevant for this topic are enzymes involved in SphL metabolism as neutral and acid sphingomyelinase (nSMase, aSMase), ceramidase (Cerase), sphingosine kinase (SphK), glucocerebrosidases 1 and 2 (GBA1 and GBA2) and galactosylceramidase (GalCerase), and glucosylceramide synthase (GCerS) (Figure 1).

SM is hydrolyzed by SMase to produce phosphocholine and Cer. The SM breakdown is carried out by different SMase isoenzymes belonging to three families classified based on their activity pH optima into acid, and neutral subtypes: aSMase and nSMase (2). aSMase that is specifically located in lysosomes and under stress conditions, rapidly translocates from lysosomes to the outer leaflet of the plasma membrane (2). An array of nSMases reside in specific subcellular structures, including cell nucleus (3). aSMase is encoded by *SMPD1* gene and the four nSMase isoforms are encoded by different genes, nSMase1 by *SMPD2*, nSMase2 by *SMPD3*, nSMase3 by *SMPD4*, and MA-nSMase (mitochondrial-associated nSMase by *SMPD5* (4).

In this review, we provide an overview on the changes and roles of SphLs in Alzheimer's disease, Parkinson's disease and Amyotrophic lateral sclerosis, and on the possibility of being interesting molecules as diagnostic markers and therapeutic targets.

## Sphingolipids in Storage Disorders as a Cause of Neurodegeneration

Disorders of sphingolipids metabolism in lysosomes induced a family diseases identified as lysosomal storage diseases (LSDs). LSDs include Niemann-Pick disease (NP), Gaucher's disease (GD), Farber's disease (FD) and Krabbe's disease (KD) (Table 1).

**Table 1:** Lysosomal storage diseases caused by impairments in sphingolipid metabolism.

**Type of disease**	**Accumulated compound**	**Deficient enzyme**
Krabbe disease, galactosylceramide lipidosis	Galactosylceramide	β-Galactosidase
Niemann-Pick disease, sphingomyelin lipidosis	Sphingomyelin	Sphingomyelinase
Gaucher disease, glucosylceramide lipidosis	Glucosylceramide	β-Glucocerebrosidase
Fabry disease, trihexosylceramidase lipidosis	Trihexosylceramide	α-Galactosidase
Farber disease, ceramidase deficiency	Ceramide	Ceramidase

NP type A and B (NPA-NPB) is characterized by defective activities of aSMase resulting in defect of SM degradation. SM cannot be converted to Cer and consequently Cer-SM ratio is altered. It means that signal cascades with participation of ceramide is not developed (5, 6).

GD is caused by the absence of GBA leading to the accumulation of the glucosylceramide (GCer) and glucosylsphingosine (GSph). GCer and GSph accumulate in mononuclear phagocytes (due to the phagocytic activity of these cells), primarily macrophages, which have a foamy appearance and are referred as Gaucher cells (7–10). In the brain, accumulation of GSph causes neuronal storage of gangliosides leading to loss of neurons and their axons, resulting in cortical atrophy and white matter degeneration (11).

FD is characterized by the deficient activity of acid ceramidase (aCerase) and high levels of Cer (12). The rate of Cer synthesis in brain during FD is normal but Cer resulting from degradation of complex SphLs cannot be hydrolyzed and accumulated into lysosomal compartment. The abnormal Cer level in the brain results in neuronal dysfunction (13, 14).

KD is caused by the deficiency of galactosylceramidase (GalCerase) in lysosomas which remove galactose from galactoceramide derivatives. The disease is due to mutations in the GALC gene (14q31) encoding the lysosomal enzyme GalCerase, that catabolizes the hydrolysis of galactose from galactocerebroside (GalCer) and galactosylsphingosine (GalSph) or psychosine. The accumulation of cytotoxic psychosine leads to apoptosis of oligodendrocytes and demyelination of the CNS and PNS. Rarely, infantile KD is caused by a mutation in the prosaposin (PSAP) gene (10q21-q22), encoding SL activator protein saposin-A, necessary for GalCerase activity (15).

## Sphingolipids in Neurodegenerative Disorders

Neurodegenerative disorders (NDDs) include specific diseases characterized by a complex pattern of pathological hallmarks including neuroinflammation with progressive loss of structure and/or function of neuronal cells leading to a set of incurable and debilitating conditions with sometimes different and sometimes overlapping symptoms as functional decline of cognition and/or movement (16). The main neurodegenerative diseases are Alzheimer's disease (AD), Parkinson's disease (PD), and Multiple Sclerosis (MS). Many SphL species, as shown so far in few studies, undergo significant changes during NDDs. The important role of SphLs in neurodegeneration is easily understood by looking at the effects induced by their accumulation. These diseases are characterized by a combination of axonal, neuronal, and myelin defects, in addition to astrogliosis, and neuroinflammation and therefore neurodegeneration (17). It is evident from genetic analysis that mutation affecting lipid manipulating enzymes impact on lipid mediators activities, lipid transport and on important cell functions, such as autophagy and inflammation, thus acting as an independent risk factor for age-related neurodegeneration (18, 19).

### Alzheimer's Disease

AD is the most common cause dementia in adults over 65 years old (20). The number of AD patients will increase over 80 million people by 2040 (21). Despite significant efforts undertaken by the international community, precise mechanism of the development of AD still remains unknown. In contrast to other cognitive pathologies causing clinical signs of dementia, the cardinal pathologic features of AD are amyloid beta (Aβ) aggregates, the major components of senile plaques, and neurofibrillary tangles of τ protein (22). It has been shown that amyloidogenesis plays a key role in induction and development of AD (23). Aβ with molecular mass of 4 kDa was originally described in 1985 (24). It is formed from proteolytic cleavage of a constitutive transmembrane protein, Aβ precursor protein (APP), catalyzed by β- and γ-secretases that are at the heart of the AD pathogenesis even if recently also a δ-secretase has been identified (23). Alternative APP cleavage by α-secretase does not result in amyloid peptide formation (25).

#### Sphingolipids in the Brain

During the last two decades much attention was paid to brain sphingolipids (SphLs) in neurodegeneration (26–28) as molecules involved in the processing and aggregation of Aβ, in the signal transduction of a cytotoxic signal induced by Aβ and of pro-inflammatory signal induced by cytokine TNFα, that are considered the main inducers of AD (29). Neuronal plasma membrane is a primary target for Aβ and its lipid component is directly involved in Aβ neurotoxicity mechanisms. The most important conformational changes of Aβ occur in the presence of SphLs. V3-like domain of Aβ interacts with SM and GalCer in monomolecular films at the air-water interface (30). Such changes in the membrane composition might also influence activity of enzymes involved in APP processing.

In a study of vesicle models to mimic exosomes, GM1 promotes Aβ fibril formation (31). It has been suggested that formation of such structures is a prerequisite for subsequent formation of Aβ plaques (30, 32). Identification of factors resulting in monosialic GM (GM1)/Aβ complex formation would represent a novel mechanism of the pathogenesis of AD and give a possibility for development of a novel strategy for prevention and treatment of this disease.

It has been demonstrated that rafts, special plasma membrane domains enriched in GSphLs, cholesterol (CHO), SphLs, and membrane proteins involved in extracellular signal transduction play a specific role in Aβ aggregation (33, 34). APP is transported and proteolytically cleaved by β-, and γ-secretases located in CHO-SM domains (34). Thus, lipid composition of rafts including GSphLs, CHO, SM and its metabolites, Cers, may strictly control APP processing and Aβ aggregation.

##### Neutral and acid sphingomyelinase

A soluble Aβ oligomer causes activation of both nSMase and aSMase. Specific inhibition and knockdown of each enzyme provides cell resistance to Aβ- induced apoptosis (35).

In primary neuronal cells the treatment with fibrillar Aβ causes nSMase upregulation and consequently the increase in Cer content (35). Inhibition of nSMase decreases cell death thus suggesting that nSMase is essential for Aβ cytotoxicity (35). The intracerebral administration of Aβ or TNFα to rats results in nSMase activation. This process is more pronounced in hippocampus than in cortex and cerebellum (29). He et al. (27) provided evidence on aSMase role in Aβ cytotoxicity.

Astrocytes, the main representative of glial cells, activated by 1 μM Aβ-142 in combination with 10 ng/ml IL1β were used in studies of mechanisms of their toxic effect on human primary neurons (36). These studies demonstrated a sharp activation of nSMase and Cer accumulation in neurons during their death induced by astroglia activation. Some studies revealed association of the SM cycle signaling system with oxidative stress (29). It was found that reactive oxygen species directly influence SMase or other enzymes involved in regulation of SM metabolism. This may potentiate the toxic effect of cytokines and Aβ on the brain cells during combined action of these compounds. Successful employment of antioxidant therapy in clinical practice confirms effective inhibition of processes related to activation of oxidative systems in the development of AD. The relationship between nSMase and nitric oxide synthase (NO) is controversial. In fact, it has been described that activation of neuronal nSMase is determined by NO generated by activated astroglia. Otherwise, Kumar et al. (37) found that in activated astrocytes nSMase induces mRNA expression of inducible nitric oxide synthase (iNOS) together to an overexpression of pro-inflammatory cytokines (TNFα, IL1β, IL6). Involvement of Aβ in Cer generation in neurons was demonstrated showing that only Aβ142 but not its reverse form (Aβ42–1) induces nSMase activation (38).

In oligodendrocytes, Aβ induces apoptosis accompanied by the increase in the Cer level (39). Addition of exogenous Cer or bacterial nSMase, by increasing the Cer level, to the oligodendrocyte cell culture causes cell death (38, 39). nSMase inhibitor (3methylsphingomyelin) effectively protects oligodendrocytes against Aβ effects (39). In these cells, Aβ influences expression of iNOS induced by TNFα through nSMase/Cer pathway (40). This molecular relationship is fundamental. In fact, neither Cer nor Aβ alone cause iNOS expression that instead is stimulated only by accumulation of TNFα. On the other hand Aβ is only able to activate nSMase. This is relevant for understanding the mechanism of AD development, in which pro-inflammatory components play important roles (40). It should be noted that the natural antioxidant glutathione inhibits nSMase activity. A decrease in the glutathione level causes activation of this enzyme and accumulation of Cer in oligodendrocytes followed by their death (39).

In dendritic cells, the effect of Aβ results in aSMase activation and its inhibition results in the resistance of these cells to Aβ-induced apoptosis (41). A study of postmortem analysis of the AD patient brains revealed activation only of aSMase (38).

Taken together, the above studies suggest the involvement of different SMase forms in the realization of Aβ-induced apoptosis of nerve cells. Analysis of genes encoding aSMase and nSMase2 expression revealed their upregulation in the brain of patients with both AD and other neuro pathologies (42). Since SMase activation was investigated in various cell lines, during actions of various forms of Aβ, in animal experiments and in studies of human postmortem brain preparations, discrepancies in results obtained in different systems is not surprising. There is no reasonable transgenic animal model which would completely mimick the pathological mechanism of AD; still remains unclear which type of Aβ is responsible for cell death and loss of cognitive functions in humans. Although local concentrations of fibrillar Aβ found in the brain of AD patients may differ from Aβ concentrations in primary neurons used in experiments, the data on interrelationship between SMases, Cer accumulation and subsequent death of neurons and oligodendrocytes suggest that SMases may be perspective targets for drugs preventing neurodegenerative impairments in AD. Thus, induction of the SM cascade cycle resulting in accumulation of the proapoptotic agent Cer may be considered as a novel mechanism of the development of AD. This may be considered as a prerequisite for novel approaches to AD therapy by using drugs of a new generation that inhibit SMase activity (43–45).

##### Ceramides

Cer accumulation is evident in early stages of AD disease while the later stages are characterized by decreased Cer levels in brain structures (46–48). In particular, the authors found in later stages of AD decreased levels of Cer in the white matter of the middle frontal gyrus in AD patients compared with corresponding controls (46). Analysis, performed in early stages of AD and in other brain impairments, of six Cer species, differing on the basis of fatty acid chains, revealed increased levels of Cer16:0, Cer18:0, Cer20:0, and Cer24:0 in comparison with controls (42). High ceramide levels are responsible for the increased susceptibility of neurons and oligodendrocytes to cell death (49). Interesting results were obtained with the gene expression studies for enzymes involved in the control of synthesis and degradation of Cer during the development and course of AD (48). It was firmly recognized that the early stage of AD is characterized by increased level of Cer *de novo* synthesis, by stimulation of Cer synthases, especially of Cer containing C22:0 and C24:0 long chain fatty acids, while synthesis of GCer decreases (48). These results support above reported studies indicating Cer accumulation in early stages of AD and therefore Cer synthase might by an early target for reducing AD progression.

##### Sphingosine and sphingosine-1-phosphate

It has been shown an accumulation of Sph, with proapoptotic properties, during a post mortem study of AD patient brains (27). In support, the activity of aCerase converting Cer to Sph is higher in AD patients compared with controls (27, 50, 51).

In contrast to Cer and Sph, S1P, a product of Sph phosphorylation, exhibits antiapoptotic properties and it is involved in the regulation of cell proliferation (27). There are data demonstrating S1P reduction in the cytosolic fraction of the gray matter of the frontotemporal brain region of AD patients compared with controls (27). There is negative correlation between S1P and Aβ and phosphorylated τ protein in the same brain region (27).

**Figure 2:**
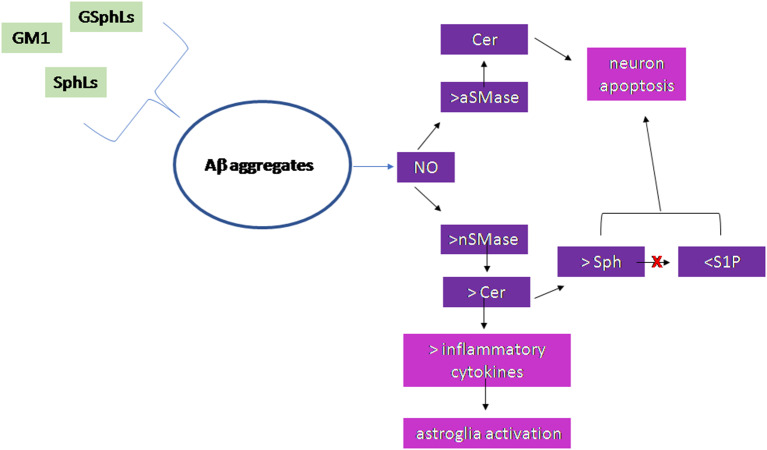
Scheme of the sphingolipid involvement in Alzheimer's Disease. Glycosphingolipids (GSphLs), *ganglioside 1* (GM1), sphingolipids (SphLs) are involved in the formation of Aβ aggregates. Aggregates stimulate nitric oxide that induces activation of both acid sphingomyelinase (aSMase) and neutral sphingomyelinase (nSMase). The increase of aSMase activity is responsible for ceramide (Cer) production in lysosomes that induces neuron apoptosis. The increase of nSMase activity produces cell membrane Cer that either is able to stimulate the synthesis of inflammatory cytokines with consequent astroglia activation or is catabolized to sphingosine (Sph). The sphingosine kinase is inhibited and therefore sphingosine-1-phospahte (S1P) level is very low. Both the accumulation of Sph and the reduction of S1P are responsible for neuron apoptosis.

Figure 2 summarizes the results of the research work carried out on the brain.

#### Sphingolipids in Biofluids

Some researchers believe that correlation between changes of cerebrospinal fluid (CSF) and blood SphL species in AD patients imply the applicability of SphLs as AD biomarkers, especially at the early stage of this disease, and as putative targets for novel drug generation (52, 53).

##### Sphingolipids in the liquor

CSF can most adequately reflect the brain pathological changes in AD patients. Usually, changes in the brain lipids are studied in AD patients at the terminal stage of this disease, while studies in CSF lipids may monitor development of the disease and effectiveness of treatment. Analysis of CSF revealed that SM species increase at the early stage of this disease, while no changes are found in AD patients at the mid and severe stages (53). In AD patients, CSF Cer content is significantly higher than that present in other neurological diseases as cervical spondylosis, lateral amyotrophic sclerosis, metabolic encephalopathy, stroke (54). The highest Cer total content is found in the mid stage of AD compared with early and severe stages (52). Nevertheless, the use of Cer family components as AD markers has been widely discussed in the literature (52–54).

Although the change of Cer species is not specific for AD but it is present also in other neurological pathologies, it may be used as a marker of a particular stage of AD. Unlike the total content of Cer, changes in the Cer species may indicate early impairments in AD, which, possibly, may be responsive to therapeutic correction (52). These results suggest that impairments in SphL metabolism might be useful as a risk marker for the development of AD (53).

##### Sphingolipids in the blood

At the early stage of AD Cer content decreases in the blood, and increases in the brain and CSF (52, 53). Relationship between changes of plasma SphLs and cognitive impairments has been intensively studied in AD patients for predicting of the rate of the development of dementia (52, 53). Mielke et al. (55) studied the content of Cer, DHCer, SM and DHSM in plasma of 120 patients with AD dementia and dementias associated with other neuropathologies. During the period of more than 2 years of observation, authors found an increase in Cer and DHCer associated with rapidly progressive dementia. In patients characterized by increased levels of SM, DHSM, SM/Cer, and DHSM/DHCer ratio, dementia was slowly progressive. The authors also demonstrated that changes in CHO and triglyceride levels were not associated with the rate of dementia. This suggests that the increase in the ratio SM/Cer and DHSM/DHCer in blood of AD patients may be a predictive marker for the rate of the development of this disease. Similar results have been obtained during mass spectrometry analysis of plasma lipids in 26 AD patients and 26 elderly patients with normal cognitive functions (56). Among 33 SM species, the content of eight molecular species containing fatty acid aliphatic chains of 22 and 24 carbon atoms was significantly lower in AD patients compared with control. At the same time, the plasma levels of two Cers, C16:0 and C21:0, were significantly higher in AD patients, while the increase of other 5 Cers was not significant. The ratio of Cers/SMs containing identical fatty acids sharply differed in AD patients compared with normal controls. These changes reflected impairments of cognitive functions in AD patients. Therefore, SphL assay would be useful for monitoring the AD progression. The latter is especially important when patients are treated by novel drugs or novel drugs are under preclinical or early clinical trials.

### Parkinson's Disease

PD is the second most common neurodegenerative disease, prevalent in men, without differences for ethnicity, that affects ~1.5 to 2.0% of people over 60 years old and 4% of people over 80 years old (57). PD is due to the dopamine synthesizing neurons in the substantia nigra degeneration resulting in the decline of neurotransmitter dopamine level in the striatum with consequent movement disorders. The common manifestations are slowness and difficulty with dexterity, tremor, slow walk, unsteadiness and falls, low vocal volume, facial hypomimia (58). Prior to the movement disorders or emerging with motor disease progression may appear non-motor features as cognitive decline, including dementia, constipation, hyposmia/anosmia, depression, anxiety, and other neuropsychiatric features, sexual dysfunction, sleep complaints, urinary frequency, and orthostatic hypotension (58).

The principal cell characteristic of PD is the accumulation in neurons of α-synuclein (α-syn) protein aggregates that may be present in inclusion Lewy bodies (59). α-Syn is an acidic protein of low molecular weight (14 kDa), expressed in normal brain, peripheral nervous system and circulating erythrocytes (60). 1-methyl-4-phenyl-1,2,3,6-tetrahydropyridine (MPTP), a neurotoxin used to reproduce PD in animals, induces a strong expression of e-cadherin, and variation of length and thickness of the heavy neurofilaments (61).

Few observations on simple SphLs and more on complex SphLs as molecules involved in PD pathogenetic mechanisms have been reported.

#### Sphingolipids in the Brain

##### Neutral and acid sphingomyelinase

An upregulation of iNO synthase and a downregulation of nSMase protein expression in the hippocampus of mice in which PD was induced by MPTP have been demonstrated (62). Interestingly, the administration of MPTP in Toll-Like Receptor 4 knockout mice causes an increase in nSMase protein expression and enzyme activity in the midbrain with a decrease in SM and an increase in Cer levels, as well as a marked delocalization of the enzyme from the cell membranes, by suggesting a possible role of TLR4 in the change of SM metabolism during MPTP neurotoxicity (63). The authors demonstrate that exposure of Toll-Like Receptor 4-deficient mice to MPTP reduces unsaturated SM species by increasing saturated/unsaturated SM ratio. Since saturated fatty acid make SM a more rigid molecule in the membrane, the change of SM species might contribute to reduce neural plasticity (63). Different role has the aSMase. Interestingly, the well-known antidepressant desipramine, which is used in the clinic to treat depression in patients with Parkinson's Disease (PD), is an inhibitor of aSMase (64). Thus, it is necessary to look for new promising drugs for the treatment of PD among aSMase inhibitors that will block the toxic signals of pro-inflammatory cytokines, protecting dopaminergic neurons from death therefore will reduce cognitive impairment.

##### Ceramide, sphingosine, and sphingosine-1-phosphate

Disturbances in the Cer metabolism are associated with the appearance of Levy bodies (65). Interestingly, it has been reported by Paciotti et al. (66) that inhibition of aCerase by carmofur in *GBA*-PD derived dopaminergic neurons, resulted in lower levels of α-synuclein, possibly because of its enhanced degradation by Cer activated Cathepsin D. Unlike the proapoptotic SphLs, as Cer and Sph, S1P protects cells from apoptosis. In fact, the addition of exogenous S1P to the culture medium led to an increase in cell survival. Probably, it could perform the same function in PD. Inhibition of SphK causes an increase in synuclein secretion and activation of proapoptotic genes, such as Bcl2 family genes such as Bax (67). Thus, inhibition of the activity of SphK1 responsible for the synthesis of S1P, leads to apoptosis of neuronal cells simulating PD. It is believed that the lipid composition of lipid rafts, including GM, SM, CHO, Cers can strictly control the processing of α-syn and its aggregation (68). More detailed characteristics of rafts, identified at different stages of PD, could serve to diagnose and prevent the disease.

##### Glucosylceramides and galactosylceramides

Disorders of SphLs in the study of PD mechanisms has been associated with the development of symptoms of this disease in some types of Gaucher disease (GD) (69, 70).

The main trigger of PD is the presence of mutations in the GBA gene encoding the GCase enzyme, which cleaves GC to glucose and ceramide. The two most common mutations were identified in patients of all ethnic groups (71). Although some representatives have a greater variety of mutations, reaching up to 8. In 2009, an unprecedented international study of mutations in the GBA gene was conducted in 16 centers from 12 countries, including 5,691 patients and 4,898 controls. The obtained data gave grounds to determine a direct relationship between mutations in GD and PD. The presence of mutations in the GBA gene is typical for both familial and idiopathic forms of PD. Since a close relationship between PD and mutations in the GBA gene has been reliably established, studies began in 2010 on the changes in SphLs that are the substrate of this enzyme, primarily GC. A decrease in the GCase activity leads to dysfunction of lysosome with the accumulation of GC inside, that is responsible for the stabilization of oligomeric aggregated toxic form of α-Syn with consequent neuronal cell death (72). Decreased activity of GCerase was found in the substantia nigra and frontal cortex of patients with PD compared to controls (73). Therefore, an increase in GCerase activity is currently being considered as a new therapeutic strategy in the treatment of synucleinopathies, including PD (74). Although most studies focused on the role of GCase in PD, it has been shown that not only GCase is involved in the pathogenesis of disease but also its metabolic products, such as GSph, which has a high toxic effect on neurons (75).

The authors showed that GSph content in GD is even higher than GCer. However, role of this metabolite has not been studied for PD yet. This study gives indications on GSph as potential more striking marker of PD.

From all studies above reported, it is evident that the changes of SphLs in the initial stages of PD are accompanied by an increased risk of developing the disease with consequent dementia complications. The correction of Sph specific specie levels by regulating the activity of enzymes involved in its metabolism can either slow down or prevent the development of pathology. Such studies will certainly make it possible to discover new targets among the enzymes of Sph metabolism and to create new drugs for the prevention and treatment of PD.

**Figure 3:**
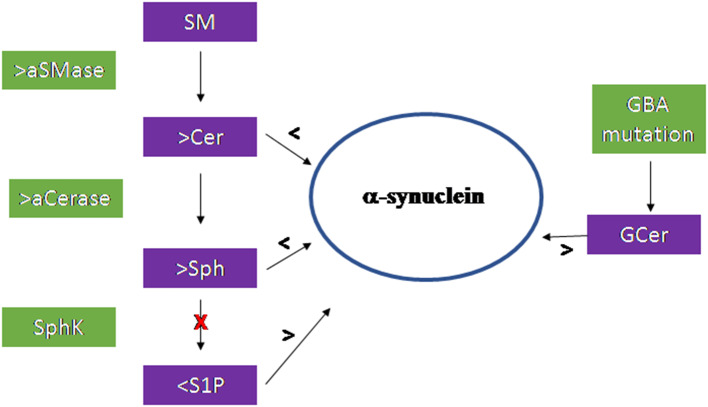
Scheme of the sphingolipid involvement in Parkinson's Disease. Acid sphingomyelinase (aSMase) and acid ceramidase (aCerase), by producing ceramide (Cer) and sphingosine (Sph), respectively in lysosomes, are responsible for the reduction of α-synuclein production and aggregation. The inhibition of sphingosine kinase (SphK) reduces sphingosine-1-phospahte (S1P) level with consequent increase of α-synuclein. Mutation of the GBA gene encoding the glucocerebrosidase enzyme induce accumulation of glucosylceramide (GCer) that induces α-synuclein increase.

Figure 3 summarizes the results of the research work carried out on the brain.

#### Sphingolipids in Biofluids

Metabolic changes in the sphingolipid system in the initial stages of PD are accompanied by an increased risk of developing this disease with dementia complications, and the correction of the level of certain types of sphingolipids by regulating the activity of enzymes involved in their metabolism can either slow down or prevent the development of this pathology. Such studies will certainly make it possible to discover new targets among the enzymes of sphingolipid metabolism and create new drugs for the prevention and treatment of Parkinson's disease. Fingolimod (FTY720), which is a structural analog of sphingosine and interacts with the S1P1 receptor, can serve as such an example. Treatment with FTY720 attenuates motor deficit and prevents dopaminergic neuronal loss in two mouse models of PD (76).

##### Sphingolipids in the liquor

β-GCase activity in CSF is reduced in PD patients independent on GBA1 mutation (77).

##### Sphingolipids in the blood

In plasma Sph changes in PD patients are associated with Lewy body identified in the autopsy sample of the brain (74). Mielke et al. (78) demonstrated that in PD patients the plasma levels of total Cer, monohexosylceramides, and LCer and those of their molecular species, with fatty acid chain from 16 to 26 carbon atoms, are significantly higher than those of controls. It is important to know that variations of SphLs are specific because CHO and triglycerides in low and high density lipoproteins do not differ respect to the control. Moreover, Cer species change in association with cognitive impairment. In fact, C14:0 and C24:1 Cer levels are significantly higher in PD dementia than in PD with no cognitive impairment and normal controls. In addition, verbal memory is negatively correlated with C14:0 and C24:1, and C22:0, C20:0 and C18:0 are associated with hallucinations, anxiety and sleep behavior disturbances, respectively. In addition, elevated levels of GM3 (79). and ganglioside-NANA-3 (80) are present in the plasma PD patients.

### Amyotrophic Lateral Sclerosis

Amyotrophic lateral sclerosis (ALS) is an incurable neurodegenerative disease characterized by selective degeneration of motor neurons in the spinal cord, motor cortex, and brain stem. Clinically, the disease is manifested as the muscular exhaustion, speech and swallowing impairments, fasciculation, and changes in reflexes and plasticity. The patients die 3 to 5 years after appearance of the first disease symptoms mainly because of the respiratory paralysis. The etiology of ~90% registered ALS cases is unknown and this form is classified as idiopathic. The remaining 10% are hereditary ALS forms associated mainly with autosomal dominant mutations in particular genes (81). Gene mutations associated with ALS as SOD (82), VCP (83), OPTIN (84), and UBQLN2 (85) for protein responsible for conformational instability and aggregation of specific proteins, genes as C9ORF72 (86), TDP43 (87), and FUS (88, 89) for proteins useful for the impairments in the RNA processing and transport, and genes as PFN1 (1), DCTN1 (90), and TUBA4A (91, 92) for protein responsible for changes in the cytoskeleton dynamics have been described. The outstanding genetic variability of ALS explains its complexity, when different mechanisms result in similar disease pathogenesis. The key features of ALS development are excitotoxicity, oxidative stress, dysfunction of mitochondria (85), neuroinflammatory and immune reactions (86).

Recently, apoptosis has been intensely studied as one of possible mechanisms responsible for the degeneration of motor neurons in ALS (89). In this connection, much attention has been drawn to the studies on the disorders of lipid metabolism in ALS development. Both idiopathic and hereditary forms of ALS are accompanied by lipid metabolism disorders, the most frequent of which is hyperlipidemia (91, 92). One of the characteristic ALS symptoms observed in ~66% patients is weight loss due to the hypermetabolism with disorder of lipid metabolism (91). Lipids perform the regulatory role by acting as secondary messengers in the inflammation processes during ALS development which are accompanied by the activation of microglia, loss of neuromuscular junctions, and subsequent degeneration of motor neurons. At the same time, the level of neurotoxic molecules synthesized with an active involvement of lipid messengers increases (93).

#### Sphingolipids in the Brain

Recently, it has been given special attention on the study of the involvement of SphLs in the pathogenesis of ALS in connection with the multiplicity of their functions in the structure and physiology of the brain. Changes in SphL species can be a manifestation of both ALS idiopathic and hereditary forms and affect the rate of disease development (91, 94).

##### Ceramides

Cutler et al. (95) showed that patients with idiopathic ALS and SOD1G93A transgenic mice that reproduces a model of hereditary ALS have high level of C16:0 and C24:0 Cer, and C16:0 SM in the lumbar spinal cord. Moreover, accumulation of C16:0 Cer in animals is evident even at the pre-asymptomatic stage of ALS. These changes are not present in the cervical spinal cord of transgenic mice, indicating the vulnerability of the lower motor neurons (96). The authors also showed that oxidative stress, which is an early event in the development of ALS can be changed by Cer.

In a subsequent study, Dodge et al. (97) demonstrated a significant increase in the total content of Cer and in its C18:0, C24:1, and C24:0 molecular species in samples of the gray and white matter of the cervical spinal cord of patients with idiopathic ALS. An increase in the Cer content is not associated with a decrease in the activity of enzymes that mediate its degradation which is typical for a group of diseases with impaired lysosomal metabolism (95).

The activity of enzymes responsible for the formation of Cer increases at different pH values. Glucocerebosidase 1 (GCBase1) activity increases at acidic pH values, GCBase2, and GalCerase activity increases at neutral pH values, which indicates the possibility of intensification of SphL hydrolysis both in lysosomes and in the plasma membrane of the cell (97). In the motor neurons of ALS patients, a predominant formation of Cer by catabolic pathways and not as a result of *de novo* synthesis was demonstrated (98). In opposition, it has been shown that apoptosis of motor neurons in SOD1G93A transgenic mice that reproduce ALS is accompanied by the generation of Cer due to the activation of the neutral SMase enzyme (98). The change in the content of Cer and other SphLs in the lumbar spinal cord of transgenic mice essentially depends on the stage of ALS. In the earlier stages of the disease, the level of C24:1 Cer and that of the most complex glycosphingolipids are lowered, and in the terminal stage of disease the level of C24:0 Cer shows an insignificant increase compared to the control (95). However, in the earlier stages of the disease, the level of C24:1 Cer and most complex glycosphingolipids is lowered.

##### Sphinganine, sphingosine, and sphingosine-1-phoshate

Sphingoid bases—sphinganin (Sphn) and Sph have bright proapoptotic properties. The proapoptotic effect is associated with the ability of these SphLs to interact with DNA, affect the activity of replication and transcription enzymes, the regulation of transcription factors and topoisomerases (47, 99, 100). SphK, that lead to the synthesis of anti-apoptotic S1P, reduces Sph pool thereby saving neuronal cells from death. A pronounced dysregulation in the metabolism of sphingoid bases, including Sphn, Sph, and S1P, in transgenic FUS mice (1-359) simulating ASL, was demonstrated (101). The study provide evidence that Sphn and Sphincrease sharply mainly in the spinal cord of mice, while their content is low and practically does not change in brain structures during the development of ALS. The ratio of S1P/ Sphn-Sph, decreases by indicating a sharp intensification of cell death in the spinal cord.

Significant disturbances were also found in the expression of SphL metabolism genes at different stages of ALS, mainly in the spinal cord. The gene expression of acylsphingosine amidohydrolase 1 (Asah1) and acid Cerase (aCerase), localized in lysosomes, increases while the expression level of Asah2 and neutral Cerase (nCerase), located on the surface of the plasma membrane, significantly decreases during the progression of FUS-mediated proteinopathy (101).

ASAH1 of lysosomes hydrolyzes saturated C10:0 and C14:0 Cer or unsaturated C18:1 and C18:2 Cer. Substrates of ASAH2 are C16:0 and C22:0 or C26:0 and C36:0 Cer. Thus, Sph is generated by lysosomal Cerase from Cer with shorter fatty acids. A change in the activity of lysosomal Cer may indicate the development of lysosomal apoptosis. The sharp increase in the expression of S1P lyase (S1PL) at the early stage of ALS is responsible for a decrease in anti-apoptotic reserves of motor neuron cells and the rapid development of apoptosis at the terminal stage of these disease of ALS, because S1PL degradates S1P that protects cells from apoptosis to the final products ethanolamine phosphate and hexadecenal.

##### Glucosylceramides and galactosylceramides

An analysis of the cervical homogenates of the post-mortal spinal cord of ALS patients shows an increased content of C18:0 and C24:1 GCer. Analysis of the spinal cord samples of SOD1G93A transegnic mice isolated for different periods of the disease also shows an increase in the content of the C24:1 GCer at the terminal stage of ALS (102). Henriques et al. (103) found significant changes in the composition of GCer already at the pre-symptomatic stage of ALS, and not only in the central nervous system, but also in the muscles of transgenic animals. While the levels of most GCer studied by the authors are reduced in the spinal cord of pre-symptomatic and symptomatic mice, the levels of many of GCer species are increased in muscles at the same stages of ALS.

The content of GCer and, accordingly, the first stage of biosynthesis of complex glycosphingolipids is controlled by glucosylceramide synthase (GCerS), a transmembrane protein of the Golgi complex. The level of expression of GCerS mRNA was significantly increased in muscles at the asymptomatic and symptomatic stage of ALS in SOD1G86R transgenic mice. In the spinal cord, the level of GCerS mRNA does not change, as there are no changes in the GCer content. An increase in GCerS activity, with formation of GluCer, is a known negative regulator of apoptosis Cer-induced (104) by indicating the possibility of a protective role of GluCer in ALS. The content of the C24:1 galactosylceramide (GalCer) form is increased in the white matter of the cervical spinal cord of patients with sporadic ALS.

##### Lactosylceramides

The formation of C18:0 lactosylceramide (LacCer) due to lactosylceramide (LacCerS) is observed in the spinal cord at the terminal stage of the disease in patients with ALS. This occurs against the background of increased activity of α-galactosidases, enzyme for LacCer synthesis, both at acidic and neutral pH values (105). The formation of LacCer can contribute to the development of the disease, since it is a mediator of inflammation and apoptosis (106) and activates microglia via the NF-κB signaling pathway (107), which is involved in the process of motor neuron death in ALS.

Moreover, LacCer activates microglia along the NF-κB signaling pathway (107), which is involved in the process of motor neuron death in ALS (104).

##### Gangliosides

Indications for deviations in GM homeostasis in ALS appeared in the literature at the end of the last century (108). Increased levels of single GM (109) due to ganglioside synthase (GMS), GM2 in the motor cortex (110), antibodies against GM2 and GM1 (111), were found in patients with ALS. Yim et al. (112) demonstrated that the total content of the neurotrophic GM3 is significantly increased in SOD1G93A transgenic mice, in relation to the course of disease. In support, a lipidomic study showed an increase of C18:0 and C24:1 molecular species of GCer (79), At the symptomatic stage of ALS in SOD1G86R mice, there is a significant increase in the content of GM1a in the spinal cord, and in the content of GM3 and GM2 in the muscles (113, 114). In the same animals, the amount of hexosaminidase mRNA, an enzyme that metabolizes GM2 to GM3, is increased in the motor neurons of the spinal cord, both at the asymptomatic stage and at the symptomatic stage of the disease (115). Also, in the spinal cord of mice SOD1G93A and patients with idiopathic form of ALS, hexosaminidase activity increases (116).

**Figure 4:**
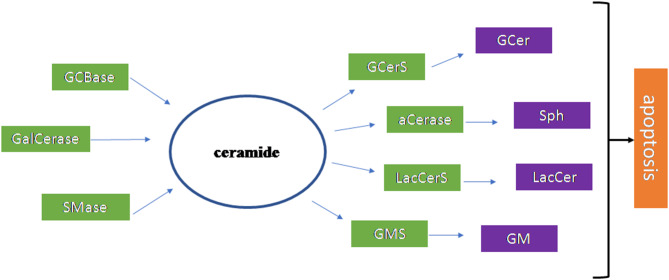
Scheme of the sphingolipid involvement in Amyotrophic Lateral Sclerosis. The ceramide (Cer) is the most important sphingolipid in Amyotrophic Lateral Sclerosis. It is produced by glucocerebrosidase (GCBase), galactosylceramidase (GalCerase), sphingomyelinase (SMase) and it is used by glucosylceramide synthase (GCerS), acid ceramidase (aCerase), lactosylceramide synthase (LacCerS), ganglioside synthase (GMS) to form glucosylceramide (GCer), sphingosine (Sph), lactosylceramide (LacCer), *ganglioside* (GM), respectively. These molecules are all involved in apoptosis that occurs in disease.

Figure 4 summarizes the results of the research work carried out on the brain.

## Conclusions

Despite many studies in the field, the exact pathogenetic mechanisms of NDD are poorly understood yet. So far, there are no efficient approaches for the NDD treatment. However, the latest studies have established that degeneration occurs via apoptotic death. Apoptosis is regulated by various interconnected pathways that eventually result in the programmed cell death. In addition to the genetic regulation, apoptosis is controlled by free radicals, death receptors, caspases, proapoptotic proteins of the Bcl2 family, inhibitors of apoptosis proteins, tumor suppressor protein p53, tumor necrosis factor-α, and many other apoptosis associated molecules. Apoptosis can be initiated by the damage to DNA, mitochondria, and lysosomal membranes. Deeper understanding of biological pathways regulating metabolism of proapoptotic and antiapoptotic SphLs in NDD disease can help to clarify pathogenetic mechanisms. Studies of SphL metabolism in the experimental animals in which NDD are induced, in the brain structure from autopsy sampling and in CSF, plasma and serum from patients affected with NDD are of great interest today. Recent studies have shown that SphLs play a decisive role in the neuronal function due to regulation of cell growth, differentiation and death in the CNS. Importantly, aSMase activation, causing accumulation of Cer proapoptotic agent, may be considered as a novel mechanism of the development of NDD as well as accumulation of S1P antiapoptotic agent may be protective for the onset and/or development of diseases. Relevantly, De Wit et al. (117) demonstrated that the in frontotemporal lobar dementia ceramide production in reactive astrocytes is independent of enhanced levels of aSMase but is due to an increase in the expression of CerS. It was observed also in AD (118). In this context it is very important to investigate changes in the SphL profile in the brain of animals with NDD-induced, and in CFS and blood of NDD patients during the course of this disease and its treatment. Analyzing various blood biomarkers in different neuropathologies, a reasonable question arises: does direct correlation between changes in the blood and loss of brain structures exist? It is possible that blood changes may be associated with those of the brain via circulation system and selective permeability of the blood brain barrier? In this connection, an accurate and informative method of analysis of the lipid profile such as mass spectrometry is of special importance. Using this technique, it is possible to analyze rapidly and accurately various lipid components in numerous biological samples using minimal quantities of biological material. Thus, it was found that SphL species might be diagnostic markers of the early stage of NDD and might be very useful to follow the development of disease.

Addictionally, SphL species could be interesting as new targets for innovative therapeutic strategies. An example of such promising pharmaceutical preparation for the ALS treatment is the Fingolimod, an immunomodulatory drug binding the S1P receptors 1, 3, 4, and 5 and playing the antiapoptotic role of the S1P (119).

In conclusion, in this review we described the association between the SL metabolism disorders and the neurodegenerative disease pathogenesis. We reported relevant studies indicating remarkable roles of different SLs in maintaining neuronal health or in inducing cell death. Despite many observations about the possible application of SL pathway molecules in diagnostic and therapy, further studies are still needed to better elucidate the specificity of each SL in the development and/or progression to different stages of neurodegenerative disorders.

## Author Contributions

AA and EA equally participated in the writing and revision of the review.

## Conflict of Interest

The authors declare that the research was conducted in the absence of any commercial or financial relationships that could be construed as a potential conflict of interest.

## Abbreviations

aCerase: acid ceramidase
aSMase: acid SMase
Asah1: acylsphingosine amidohydrolase 1
AD: Alzheimer's disease
ALS: Amyotrophic lateral sclerosis
Cer: ceramides
CHO: cholesterol
FD: Farber's disease
GalCBase: galactocerebrosidase
GalCerase: galactosylceramidase
GalCer: galactosylceramide
GalSph: galactosylsphingosine
GM: ganglioside
GMS: ganglioside synthase
GD: Gaucher disease
GCBase: glucocerebrosidases
GCer: glucosylceramide
GCerS: glucosylceramide synthase
GSph: glucosylsphingosine
GSphLs: glycosphingolipids
HEX: hexosaminidase
hCer: hexosylceramides
iNOS: inducible nitric oxide synthase
KD: Krabbe's disease
LacCer: lactosylceramide
LacCerS: lactosylceramide synthase
NDDs: neurodegenerative disorders
nCerase: neutral ceramidase
NP: Niemann-Pick disease
NO: nitric oxide synthase
PD: Parkinson's Disease
Sphn: sphinganin
SphLs: sphingolipids
SM: sphingomyelin
SMase: sphingomyelinase
Sph: sphingosine
SphK: sphingosine kinase
S1P: sphingosine-1-phosphate
S1PL: sphingosine-1- phosphate lyase.
